# Supplementary material for: Theanine, the Main Amino Acid in Tea, Prevents Stress-Induced Brain Atrophy by Modifying Early Stress Responses
Source: Nutrients. 2020 Jan 8;12(1):174. doi: 10.3390/nu12010174 (PMC7019546; doi:10.3390/nu12010174)
Supplement: Supplementary file 1 [file nutrients-12-00174-s001.pdf]

Table S1. Brain volume of each part.

Mouse	Group	Confrontation period (months)	Age (months)	Hippocampus	Corpus Callosum	Caudate Putamen	Anterior Commissure	Globus Pallidus	Internal Capsule	Thalamus	Cerebellum	Superior Colliculi	Ventricles	Hypothalamus	Inferior Colliculi	Central Gray	Neocortex	Amygdala	Olfactory Bulb	Brainstem	The Rest of midbrain	Basal Forebrain and Septum	Fimbria	Total
SAMP10	CC	0	2	24.38 ± 1.91	12.69 ± 0.88	26.64 ± 1.81	0.32 ± 0.03	2.72 ± 0.21	2.29 ± 0.20	25.84 ± 2.35	58.48 ± 4.21	9.22 ± 0.82	0.66 ± 0.06	10.49 ± 0.78	6.16 ± 0.42	3.88 ± 0.23	128.33 ± 6.52	11.64 ± 0.78	25.16 ± 1.26	44.79 ± 2.37	11.46 ± 0.73	12.51 ± 1.00	2.16 ± 0.16	419.81 ± 23.54
		1	3	22.91 ± 1.50	11.84 ± 0.86	23.55 ± 2.16	0.32 ± 0.03	2.45 ± 0.24	2.18 ± 0.14	23.34 ± 2.15	52.92 ± 4.96	8.04 ± 0.88	0.58 ± 0.03	10.01 ± 0.75	5.63 ± 0.51	3.37 ± 0.27	112.75 ± 8.26	10.53 ± 0.75	24.67 ± 1.33	42.71 ± 1.94	11.04 ± 0.83	11.64 ± 0.78	2.03 ± 0.08	382.41 ± 26.79
		2	4	23.76 ± 1.24	11.71 ± 0.84	24.22 ± 1.64	0.33 ± 0.03	2.50 ± 0.22	2.20 ± 0.12	23.86 ± 1.49	53.68 ± 3.92	8.45 ± 0.79	0.59 ± 0.04	10.09 ± 0.54	5.97 ± 0.33	3.59 ± 0.25	114.62 ± 7.94	10.72 ± 0.57	25.12 ± 1.31	44.37 ± 3.73	11.31 ± 0.53	11.78 ± 0.79	2.14 ± 0.15	391.00 ± 22.19
		4	6	23.89 ± 2.25	12.04 ± 1.33	24.30 ± 2.42	0.33 ± 0.04	2.64 ± 0.33	2.39 ± 0.26	24.41 ± 2.08	52.34 ± 5.68	8.38 ± 0.52	0.61 ± 0.06	10.23 ± 0.98	5.84 ± 0.37	3.61 ± 0.21	113.28 ± 9.87	11.15 ± 0.94	25.85 ± 1.04	47.19 ± 2.70	11.38 ± 0.60	11.84 ± 1.34	2.16 ± 0.13	393.84 ± 32.12
		6	8	23.01 ± 0.79	11.11 ± 0.53	22.30 ± 1.22	0.29 ± 0.02	2.50 ± 0.13	2.24 ± 0.09	23.49 ± 1.27	50.00 ± 2.95	8.16 ± 0.71	0.54 ± 0.03	9.90 ± 0.33	5.61 ± 0.31	3.47 ± 0.16	106.44 ± 5.29	10.56 ± 0.45	25.25 ± 0.91	42.56 ± 1.23	11.28 ± 0.44	11.05 ± 0.38	1.97 ± 0.09	371.72 ± 14.74
	CT	0	2	24.52 ± 0.92	13.31 ± 0.52	28.01 ± 0.92	0.33 ± 0.02	2.96 ± 0.10	2.43 ± 0.09	26.89 ± 1.09	60.07 ± 1.90	9.12 ± 0.32	0.68 ± 0.02	10.72 ± 0.67	6.28 ± 0.23	3.86 ± 0.10	131.08 ± 4.49	11.92 ± 0.45	25.46 ± 1.15	45.21 ± 1.32	11.99 ± 0.44	13.06 ± 0.69	2.20 ± 0.09	430.10 ± 11.18
		1	3	23.85 ± 1.32	12.19 ± 0.83	24.66 ± 1.72	0.33 ± 0.02	2.63 ± 0.25	2.27 ± 0.17	25.13 ± 1.97	52.18 ± 3.06	8.69 ± 0.69	0.63 ± 0.04	10.53 ± 0.69	5.87 ± 0.37	3.64 ± 0.17	114.56 ± 6.65	10.73 ± 0.37	24.85 ± 0.99	43.62 ± 1.37	11.54 ± 0.73	12.00 ± 0.91	2.12 ± 0.13	392.03 ± 19.04
		2	4	25.53 ± 1.87	12.80 ± 0.79	26.11 ± 1.56	0.35 ± 0.03	2.82 ± 0.18	2.47 ± 0.12	25.73 ± 1.65	57.13 ± 2.84	9.00 ± 0.67	0.63 ± 0.04	10.67 ± 0.63	6.30 ± 0.43	3.72 ± 0.30	123.75 ± 7.57	11.66 ± 0.93	26.28 ± 1.24	46.52 ± 2.43	12.01 ± 0.61	12.55 ± 0.82	2.23 ± 0.14	418.24 ± 21.25
		4	6	24.69 ± 1.57	12.79 ± 1.36	25.42 ± 2.68	0.33 ± 0.04	2.79 ± 0.38	2.50 ± 0.32	25.64 ± 2.41	54.59 ± 4.54	8.72 ± 0.42	0.67 ± 0.06	10.70 ± 1.04	5.96 ± 0.29	3.79 ± 0.37	117.36 ± 7.83	11.49 ± 0.90	25.89 ± 0.93	47.23 ± 2.32	11.55 ± 0.65	12.27 ± 1.76	2.35 ± 0.22	407.38 ± 28.99
		6	8	26.02 ± 1.46	12.16 ± 0.61	24.95 ± 1.87	0.33 ± 0.02	2.71 ± 0.24	2.44 ± 0.14	25.31 ± 1.58	53.76 ± 4.36	8.56 ± 0.56	0.59 ± 0.04	10.98 ± 0.82	5.78 ± 0.40	3.57 ± 0.18	114.54 ± 6.99	11.46 ± 0.53	26.39 ± 0.96	44.87 ± 1.81	11.78 ± 0.29	12.64 ± 1.05	2.21 ± 0.10	401.07 ± 20.86
	GC		2	25.35 ± 0.56	13.19 ± 0.23	27.51 ± 0.66	0.34 ± 0.01	2.88 ± 0.22	2.39 ± 0.17	27.54 ± 1.08	57.95 ± 5.26	9.32 ± 0.26	0.67 ± 0.03	11.50 ± 0.45	6.41 ± 0.16	3.85 ± 0.22	129.09 ± 3.17	11.62 ± 0.71	25.59 ± 0.94	44.26 ± 0.63	11.87 ± 0.10	13.46 ± 0.46	2.23 ± 0.12	427.12 ± 10.82
			3	24.71 ± 1.37	12.39 ± 0.73	24.83 ± 1.73	0.34 ± 0.03	2.57 ± 0.18	2.27 ± 0.12	24.34 ± 1.53	52.61 ± 2.78	8.19 ± 0.53	0.60 ± 0.04	10.38 ± 0.66	5.74 ± 0.35	3.47 ± 0.17	115.18 ± 6.77	11.13 ± 0.65	25.38 ± 1.40	42.71 ± 0.82	11.34 ± 0.45	12.27 ± 0.88	2.10 ± 0.10	392.43 ± 18.94
			4	24.38 ± 1.42	12.01 ± 0.97	24.76 ± 2.08	0.33 ± 0.03	2.59 ± 0.23	2.32 ± 0.16	24.40 ± 1.81	54.76 ± 3.30	8.32 ± 0.68	0.61 ± 0.06	10.36 ± 0.75	5.89 ± 0.39	3.48 ± 0.18	115.20 ± 8.11	11.00 ± 0.93	25.59 ± 1.32	44.23 ± 1.52	11.44 ± 0.68	12.05 ± 0.96	2.16 ± 0.22	396.41± 23.78
			6	27.03	12.05	25.15	0.35	2.62	2.42	25.88	54.83	8.69	0.62	10.41	5.89	3.76	114.82	11.72	26.91	45.14	11.75	12.33	2.32	404.67
			8	25.75 ± 1.69	11.91 ± 0.73	23.71 ± 0.42	0.32 ± 0.02	2.61 ± 0.07	2.41 ± 0.04	24.29 ± 0.87	53.39 ± 2.89	8.42 ± 0.53	0.56 ± 0.04	10.37 ± 0.30	5.86 ± 0.36	3.54 ± 0.21	110.92 ± 3.91	11.34 ± 0.43	26.43 ± 0.57	44.14 ± 1.37	11.77 ± 0.34	11.81 ± 0.36	2.10 ± 0.12	391.66 ± 9.87
	GT		2	23.45 ± 1.17	11.94 ± 1.22	25.33 ± 2.37	0.31 ± 0.03	2.57 ± 0.25	2.13 ± 0.13	24.63 ± 2.23	55.79 ± 3.53	8.78 ± 1.15	0.64 ± 0.06	10.03 ± 0.72	5.99 ± 0.50	3.77 ± 0.24	118.90 ± 11.03	11.21 ± 0.71	24.37 ± 1.18	45.86 ± 2.87	11.42 ± 0.74	12.05 ± 0.90	2.09 ± 0.13	401.25 ± 29.11
			3	24.25 ± 1.02	12.44 ± 0.97	24.72 ± 2.03	0.34 ± 0.03	2.55 ± 0.24	2.27 ± 0.13	24.27 ± 1.71	52.49 ± 3.40	8.68 ± 1.07	0.62 ± 0.06	10.32 ± 0.49	5.85 ± 0.40	3.56 ± 0.29	115.03 ± 6.99	10.97 ± 0.52	24.51 ± 0.94	45.16 ± 2.03	11.48 ± 0.68	12.01 ± 0.61	2.12 ± 0.11	393.62 ± 22.14
			4	25.91 ± 1.87	12.29 ± 0.64	24.92 ± 1.42	0.32 ± 0.02	2.59 ± 0.17	2.36 ± 0.15	24.60 ± 1.59	56.60 ± 4.97	8.51 ± 0.75	0.64 ± 0.04	10.33 ± 0.49	6.04 ± 0.42	3.62 ± 0.17	114.97 ± 8.36	10.94 ± 0.60	25.00 ± 1.28	44.14 ± 1.73	11.38 ± 0.60	11.85 ± 0.40	2.32 ± 0.21	399.34 ± 22.99
			6	23.85	11.01	23.96	0.30	2.50	2.23	23.89	56.17	8.22	0.58	9.89	5.70	3.32	110.79	10.65	24.61	43.63	11.28	11.27	2.12	385.97
			8	25.54 ± 1.91	11.74 ± 0.62	23.59 ± 1.57	0.32 ± 0.01	2.61 ± 0.23	2.39 ± 0.15	24.00 ± 1.82	51.20 ± 2.57	8.17 ± 0.25	0.56 ± 0.02	10.38 ± 0.66	5.61 ± 0.12	3.49 ± 0.15	110.21 ± 4.39	11.07 ± 0.52	26.12 ± 1.05	43.05 ± 0.95	11.51 ± 0.36	11.99 ± 0.89	2.07 ± 0.08	385.59 ± 12.89

Mouse	Group	Confrontation period (months)	Age (months)	Hippocampus	Corpus Callosum	Caudate Putamen	Anterior Commissure	Globus Pallidus	Internal Capsule	Thalamus	Cerebellum	Superior Colliculi	Ventricles	Hypothalamus	Inferior Colliculi	Central Gray	Neocortex	Amygdala	Olfactory Bulb	Brainstem	The Rest of midbrain	Basal Forebrain and Septum	Fimbria	Total
ddY	CC	0	2	27.44 ± 1.77	12.53 ± 1.10	27.71 ± 2.58	0.36 ± 0.03	2.72 ± 0.26	2.31 ± 0.18	26.39 ± 2.09	56.66 ± 2.20	8.83 ± 0.74	0.68 ± 0.06	11.34 ± 0.68	6.02 ± 0.44	4.01 ± 0.35	129.33 ± 11.37	13.01 ± 0.68	27.92 ± 1.37	45.76 ± 2.22	12.19 ± 0.81	13.33 ± 0.91	2.50 ± 0.19	431.04 ± 26.21
		1	3	24.31 ± 2.21	10.84 ± 0.83	23.46 ± 1.57	0.29 ± 0.02	2.32 ± 0.15	2.01 ± 0.20	23.22 ± 1.70	51.99 ± 5.26	7.69 ± 0.45	0.68 ± 0.07	9.69 ± 0.64	5.18 ± 0.46	3.64 ± 0.24	114.09 ± 8.93	11.58 ± 0.88	26.62 ± 1.74	46.54 ± 3.13	10.78 ± 0.80	10.95 ± 0.78	2.38 ± 0.19	388.24 ± 27.28
		2	4	26.55 ± 1.43	11.84 ± 0.98	26.60 ± 2.31	0.35 ± 0.03	2.71 ± 0.22	2.31 ± 0.15	25.49 ± 1.40	55.40 ± 3.10	8.64 ± 0.88	0.75 ± 0.06	11.05 ± 0.30	5.92 ± 0.40	3.84 ± 0.22	118.81 ± 6.76	12.22 ± 0.15	27.53 ± 0.88	46.47 ± 2.65	12.11 ± 0.60	12.72 ± 0.65	2.61 ± 0.16	413.92 ± 20.58
		4	6	26.29 ± 2.57	11.64 ± 0.89	24.85 ± 2.56	0.31 ± 0.03	2.64 ± 0.26	2.28 ± 0.20	25.76 ± 0.92	56.86 ± 4.55	8.89 ± 0.53	0.72 ± 0.05	11.20 ± 1.00	6.02 ± 0.36	3.98 ± 0.33	117.82 ± 8.75	12.66 ± 1.21	30.52 ± 1.00	50.26 ± 2.48	12.25 ± 0.43	12.54 ± 1.34	2.64 ± 0.11	420.12 ± 24.89
		6	8	28.08 ± 2.48	12.51 ± 1.02	27.17 ± 2.97	0.37 ± 0.05	2.86 ± 0.27	2.50 ± 0.19	26.80 ± 1.28	61.16 ± 8.13	8.93 ± 0.35	0.75 ± 0.03	11.95 ± 0.84	6.09 ± 0.37	4.19 ± 0.13	124.26 ± 8.96	13.42 ± 1.43	30.54 ± 1.28	53.31 ± 1.72	12.45 ± 0.42	13.51 ± 1.69	2.64 ± 0.17	443.49 ± 30.46
	CT	0	2	27.31 ± 0.75	12.42 ± 0.58	27.39 ± 1.51	0.33 ± 0.02	2.72 ± 0.14	2.25 ± 0.06	26.89 ± 0.70	56.80 ± 1.29	8.87 ± 0.30	0.70 ± 0.02	11.27 ± 0.40	5.91 ± 0.07	4.03 ± 0.19	128.88 ± 5.20	12.71 ± 0.59	26.76 ± 1.18	45.51 ± 1.60	12.18 ± 0.22	13.09 ± 0.66	2.40 ± 0.13	428.41 ± 8.55
		1	3	25.64 ± 2.73	11.64 ± 1.53	26.36 ± 3.67	0.32 ± 0.04	2.63 ± 0.35	2.223 ± 0.30	25.99 ± 2.15	54.50 ± 5.67	8.31 ± 0.66	0.74 ± 0.08	11.16 ± 1.27	5.53 ± 0.34	3.77 ± 0.29	123.56 ± 13.26	12.74 ± 1.40	27.97 ± 1.85	49.07 ± 2.77	11.63 ± 0.85	12.59 ± 1.67	2.60 ± 0.14	418.97 ± 37.88
		2	4	27.90 ± 1.35	12.38 ± 0.84	27.31 ± 2.40	0.35 ± 0.03	2.79 ± 0.22	2.34 ± 0.11	26.80 ± 2.02	55.31 ± 3.94	9.09 ± 0.99	0.76 ± 0.07	11.11 ± 0.83	6.05 ± 0.50	4.00 ± 0.28	123.77 ± 8.13	12.48 ± 0.40	27.65 ± 1.01	46.78 ± 2.59	12.29 ± 0.91	12.89 ± 1.11	2.55 ± 0.26	424.57 ± 23.55
		4	6	27.21 ± 2.86	12.80 ± 1.38	26.92 ± 2.39	0.34 ± 0.04	2.93 ± 0.27	2.44 ± 0.20	26.71 ± 1.19	59.78 ± 6.75	8.72 ± 0.65	0.78 ± 0.03	11.97 ± 0.94	5.85 ± 0.33	3.96 ± 0.41	123,44 ± 9.46	13.02 ± 1.18	30.19 ± 1.32	51.63 ± 4.14	12.56 ± 0.69	13.16 ± 1.43	2.72 ± 0.26	437.12 ± 27.12
		6	8	27.10 ± 2.71	11.81 ± 1.39	26.00 ± 3.08	0.36 ± 0.04	2.77 ± 0.36	2.41 ± 0.28	26.08 ± 2.37	58.66 ± 8.32	8.70 ± 0.69	0.69 ± 0.07	11.47 ± 0.89	6.0 ± 0.50	4.09 ± 0.32	119.16 ± 12.81	13.16 ± 1.49	30.89 ± 2.05	54.29 ± 3.30	12.32 ± 0.83	12.85 ± 1.64	2.66 ± 0.22	431.53 ± 37.60
	GC		2	25.94 ± 1.40	11.51 ± 0.87	25.85 ± 2.01	0.32 ± 0.01	2.46 ± 0.15	2.05 ± 0.15	24.88 ± 2.22	53.48 ± 2.28	8.40 ± 0.78	0.65 ± 0.05	10.33 ± 0.57	5.75 ± 0.41	3.89 ± 0.11	123.11 ± 5.78	12.07 ± 0.73	26.18 ± 1.60	45.31 ± 2.99	11.62 ± 0.85	12.19 ± 0.60	2.17 ± 0.13	408.16 ± 21.28
			3	25.69 ± 2.51	11.18 ± 1.06	24.55 ± 2.29	0.31 ± 0.04	2.47 ± 0.25	2.11 ± 0.19	24.14 ± 1.33	52.30 ± 6.36	7.94 ± 0.48	0.70 ± 0.06	10.49 ± 0.74	5.30 ± 0.41	3.84 ± 0.15	117.25 ± 9.37	12.28 ± 0.77	28.51 ± 1.48	47.34 ± 1.88	11.28 ± 0.27	11.62 ± 1.18	2.53 ± 0.09	401.80 ± 27.04
			4	27.42 ± 1.54	12.05 ± 0.50	25.47 ± 1.52	0.34 ± 0.02	2.66 ± 0.22	2.26 ± 0.17	25.53 ± 1.37	55.66 ± 2.78	8.87 ± 0.42	0.69 ± 0.01	10.84 ± 0.36	6.02 ± 0.22	3.91 ± 0.09	120.01 ± 5.42	12.23 ± 0.95	27.42 ± 1.77	46.65 ± 1.47	11.99 ± 0.44	12.48 ± 0.79	2.54 ± 0.15	415.03 ± 14.94
			6	26.48 ± 1.25	11.95 ± 0.69	25.70 ± 2.29	0.33 ± 0.03	2.71 ± 0.25	2.25 ± 0.16	24.97 ± 1.40	55.63 ± 4.53	8.46 ± 0.53	0.75 ± 0.03	11.48 ± 0.66	5.66 ± 0.38	3.72 ± 0.29	120.24 ± 7.59	12.74 ± 0.94	30.00 ± 1.28	47.00 ± 5.39	11.55 ± 0.90	13.11 ± 1.15	2.58 ± 0.29	417.29 ± 13.19
			8	23.93 ± 1.04	10.60 ± 0.50	22.99 ± 0.91	0.32 ± 0.02	2.41 ± 0.10	2.08 ± 0.14	24.35 ± 0.95	49.62 ± 1.91	8.09 ± 0.51	0.66 ± 0.06	10.28 ± 0.49	5.39 ± 0.37	3.77 ± 0.18	108.31 ± 3.62	11.24 ± 0.45	29.06 ± 1.38	50.60 ± 2.50	11.56 ± 0.51	11.07 ± 0.43	2.55 ± 0.17	388.87 ± 13.65
	GT		2	26.65 ± 1.02	11.80 ± 0.83	26.28 ± 2.03	0.33 ± 0.02	2.54 ± 0.20	2.22 ± 0.08	25.90 ± 1.89	55.13 ± 3.39	8.67 ± 0.82	0.68 ± 0.05	10.98 ± 0.75	5.89 ± 0.57	3.87 ± 0.24	125.01 ± 6.84	12.98 ± 0.49	27.81 ± 1.13	45.92 ± 1.56	12.06 ± 0.91	12.53 ± 0.85	2.37 ± 0.18	419.59 ± 20.14
			3	26.61 ± 2.12	11.28 ± 1.22	25.00 ± 2.09	0.31 ± 0.03	2.52 ± 0.26	2.08 ± 0.21	24.76 ± 2.13	54.11 ± 4.67	8.14 ± 0.91	0.70 ± 0.07	10.22 ± 0.95	5.49 ± 0.48	3.73 ± 0.34	118.72 ± 9.38	12.35 ± 1.28	28.40 ± 1.29	47.17 ± 2.35	11.18 ± 1.07	11.82 ± 1.21	2.48 ± 0.24	405.37 ± 29.83
			4	29.25 ± 1.88	12.59 ± 1.08	28.13 ± 3.86	0.37 ± 0.05	2.89 ± 0.32	2.49 ± 0.16	28.07 ± 2.58	58.59 ± 4.22	9.00 ± 0.90	0.76 ± 0.08	11.53 ± 1.00	6.16 ± 0.63	3.94 ± 0.47	124.44 ± 9.68	12.93 ± 0.69	28.55 ± 1.30	47.01 ± 2.62	12.79 ± 1.05	13.54 ± 1.48	2.56 ± 0.21	435.58 ± 31.51
			6	27.59 ± 1.51	12.77 ± 1.10	27.22 ± 3.04	0.34 ± 0.03	2.96 ± 0.34	2.49 ± 0.24	26.65 ± 0.97	59.78 ± 6.95	8.94 ± 0.29	0.80 ± 0.05	11.92 ± 1.02	6.97 ± 0.34	3.92 ± 0.14	125.23 ± 8.21	13.35 ± 0.80	30.26 ± 0.93	52.78 ± 2.50	12.47 ± 0.38	13.41 ± 1.26	2.72 ± 0.15	441.56 ± 25.67
			8	27.81 ± 1.18	13.01 ± 1.07	28.07 ± 3.47	0.38 ± 0.05	2.94 ± 0.29	2.54 ± 0.27	27.71 ± 1.71	61.29 ± 8.11	9.56 ± 0.83	0.76 ± 0.06	11.90 ± 1.18	6.36 ± 0.74	4.27 ± 0.34	128.64 ± 10.01	13.31 ± 1.34	32.08 ± 2.41	54.84 ± 3.38	13.00 ± 1.02	13.41 ± 1.89	2.72 ± 0.28	455.40 ± 35.83
